# A Method for the Analysis of the Symmetry of Excited States from GW-BSE

**DOI:** 10.3390/ijms27021062

**Published:** 2026-01-21

**Authors:** Mohammad Maymoun, Marah Jamil Alrahamneh, Alessio Saccomani, Iogann Tolbatov, Paolo Umari

**Affiliations:** 1 Dipartimento di Fisica e Astronomia, Università di Padova, I-35131 Padova, Italy; mohammad.maymoun@unipd.it (M.M.);; 2 Department of Chemical, Physical, Mathematical and Natural Sciences, University of Sassari, I-07100 Sassari, Italy; itolbatov@uniss.it; 3 Istituto Officina dei Materiali (IOM), Consiglio Nazionale delle Ricerche (CNR), I-34149 Trieste, Italy

**Keywords:** DFT, excited states, GW-BSE

## Abstract

We present a method for analyzing the symmetries of excited states previously calculated with the popular GW-BSE approach. These are expressed through the Tamm-Dancoff approximation using the so-called batches representation. The method allows to establish how an excited state is transformed by symmetry operators as plane-reflection, proper and improper axis-rotation, point-inversions. It can also report if an excited state is eigen-state of an angular momentum operator. This permits the assignment to an irreducible representation of the underlying symmetry group and a prompt labeling of the GW-BSE states. We show results for a significant set of small molecules. Our approach can be easily extended to TD-DFT and can be used to probe the local environment of localized excitations.

## 1. Introduction

The GW-BSE approach [1,2,3,4,5] permits to evaluate excited states of molecular systems through the solution of a Bethe-Salpeter equation (BSE) combined with the GW method in turn leveraging on Green’s functions and the screened Coulomb interaction (GW). Both methods are rooted on many-body perturbation theory (MBPT) and have as a starting point single particle orbitals and energies usually obtained by density functional theory (DFT). With respect to wave-functions methods, the GW-BSE one can treat on the same foot isolated and extended (e.g., crystalline) systems retaining a favorable $O(N^{3})$ scaling [6] with respect to the generic system size *N*. For small molecules comparison with well converged coupled cluster results is possible and average errors in the order of few tens of an e*V* are registered [7] according to the applied basis set and calculation details. Although for small isolated molecules a similar degree of accuracy can be achieved with time-dependent density functional theory (TD-DFT), only GW-BSE is still fully reliable for larger and possibly periodic structures [5]. Several GW-BSE implementations have been made available based either on localized basis sets [8,9] or on plane-waves representations [6,10,11,12,13,14,15,16]. Within the latter class, two research groups proposed rigorous methods, based on the so-called resolution of the identity, that are able to rely only on an explicit description of the starting occupied DFT manifold avoiding in this way the preliminary calculation of a large number unoccupied orbitals [6,7,13,17,18,19].

The GW-BSE approach allows the evaluation of individual excited states, typically described through the Tamm-Dancoff approximation, ref. [3] together with the corresponding excitation energies, and of the entire optical response in terms of the complex dielectric function [20]. Despite the widespread adoption of GW-BSE some features are still at an initial stage of development or are still lacking. This is the case of the excited state forces defined as the gradients of the excited state energies. Although approaches have been proposed more than two decades ago [21], the quest for an effortless scheme to get atomic force *for free*, as it happens in DFT thanks to the Hellmann-Feynmann theorem, is still open [22,23].

Another capability still lacking regards the analysis of the symmetry properties of the calculated excited states. Indeed, excited states must be symmetric with respect to the symmetry operations which can be applied to the underlying atomic structure. For example, diatomic molecules are invariant for rotations around the molecular axis, for reflections about any plane containing such an axis and, in case of homo-nuclear molecules inversion about the center of the molecular axis. This implies that the calculated excited states are eigen-states of the corresponding symmetry operators if they are not degenerate or are transformed into states of the same energy if they are degenerate. From the eigenvalues of the symmetry operators for non-degenerate states or from the trace of the representation matrix of symmetry operators on a set of degenerate states we can assign the irreducible representation they belong to just looking at the correspondence with the character table of the underlying symmetry group. In turn, this allows for a prompt labeling. Although for determining these eigen-values would be sufficient to specify how symmetry operators act on BSE excited states, most studies neglect this issue altogether and just label and order the calculated states according to their energy [24]. Other studies assign the correct symmetry, looking at the symmetries of the starting DFT orbitals [25]. Unfortunately, this is not a viable option for methods which avoid the evaluation of empty DFT orbitals altogether.

Here, we illustrate a method we have devised for the immediate evaluation of the expectation values and matrix elements of symmetry operators applied to excited states approximated through the Tamm-Dancoff approximation. We have implemented this scheme within our method which avoid both unoccupied DFT orbitals and the evaluation of the entire screened Coulomb operator $\hat{W}$ [6,7]. Indeed, the method permits the evaluation of the matrix element of symmetry operator between two generic many-body states comprising excited states and the ground state. The method relies on a recent scheme we built for projecting excitons [22].

First, we will show how it can be used to label BSE excited states of small molecules. Then, we will show how it can be used to investigate the symmetry of excited states of larger molecules which are localized on region of the atomic structure exhibiting some local symmetry.

## 2. Results and Discussion

In this work, all calculations were performed with the GWL code [6,7,17,26,27,28] as implemented in the Quantum Espresso (*QE*) package [29,30] which is based on the plane-wave pseudopotentials paradigm. Furthermore, this approach precludes the GW and BSE computations from explicitly summing over empty Kohn–Sham (KS) orbitals, thereby ensuring the convergence of the calculations. Importantly, we could avoid the GW step resorting to Koopman’s complaint functionals [31,32,33,34] for the quasi-particle energies and to linear response for the application of the static screened Coulomb interaction ${\hat{W}}_{0}$ [7]. We implemented our new scheme in the BSE part of the GWL code. The symmetry operations are defined on the real space grid as this ensures faster calculations. It’s worth noting that the GWL package permits to select the density of such real space grid. For the generation of KS eigenstates, the norm-conserving Vanderbilt (ONCV) pseudopotential [35] and Perdew–Burke–Ernzerhof (PBE) functional were employed to describe the electron–core interactions and exchange correlation [36], respectively, with an energy cutoff of 70 Ry for plane wave basis set. Cubic simulation cells with lattice dimensions of 20 Bohr (for small molecules) and 20 Å (for larger molecules) were employed to prevent spurious interactions between periodic images.

### 2.1. Small Molecules

We demonstrate our method carrying out calculations on a set of small molecules: water (H_2_O), methane (CH_4_), carbon monoxide (CO), and nitrogen (N_2_). These are among the most prevalent substances in many aspects of our lives and are an important component in numerous biological and chemical processes occurring in surfaces, in the atmosphere, and in solution. We address only singlet excited states as these can be optically activated. We begin with the isolated water and methane molecules. We first optimize the geometry of these molecules. As a result, for H_2_O molecule, the bond length (O-H) and bond angle (H-O-H) are 0.9674 Å and 104.554° respectively, which agree with the experimental [37]/theoretical [38] values of 0.9572/0.969 Å and 104.5/105.5°. On the other hand, in the case of the CH_4_ molecule, the bond length (C-H) and bond angle (H-C-H) are 1.0942 Å and 109.471°.

The point group symmetries of the H_2_O and CH_4_ molecules are $C_{2v}$ and $T_{d}$, which includes four and twenty-four symmetry operators divided into four and five irreducible representations, respectively, as shown in Table 1 and Table 2. For H_2_O, beside the identity operator *E* we have the 2-fold rotation operator $C_{2}\left(z\right)$ by 180° about the z-axis along the H-O-H bisector, the $\sigma_{v}\left(xz\right)$ and $\sigma_{v^{\prime }}\left(yz\right)$ plane inversions with respect to the molecular plane and the symmetry plane perpendicular to it, respectively. The excited states of H_2_O will belong to one of the $B_{1}$, $A_{1}$, $A_{2}$, and $B_{2}$ irreducible representations.

As for H_2_O all the irreducible representations have dimension one and the excited states are not degenerate we just evaluated the expectation values of the symmetry operators in order to classify them.

In the case of CH_4_, beside the identity operator *E* we find eight 3-fold rotation operators $C_{3}$, involving 120° rotations through the body diagonals, three 2-fold rotation operators $C_{2}$, involving 180° rotations across axis passing through opposite edges, six plane inversions $\sigma_{d}$ and six improper rotations S_4_. Hence, the excited states of CH_4_ will belong to one of the $T_{1}$, $T_{2}$, E, $A_{1}$, and $A_{2}$ irreducible representations. The *A*’s are non-degenerate, while *E* and the *T*’s are doubly and triple degenerate, respectively.

The symmetry analysis of excited states for molecules H_2_O and CH_4_ has been performed and the characters of the symmetry matrices are reported in Table 3 and Table 4.

From the results in Table 3, the excited states of the H_2_O molecule are eigen-states of the $C_{2}\left(z\right)$, $\sigma_{v}\left(xz\right)$, and $\sigma_{v^{\prime }}\left(yz\right)$ symmetry operators with odd (−) or even (+) parity. The first, fourth, and seventh excited states are an eigen-states with odd (−) parity under the $C_{2}\left(z\right)$ and $\sigma_{v^{\prime }}\left(yz\right)$ operators, while they have even (+) parity under the $\sigma_{v}\left(xz\right)$ operator, respectively. The second and eighth excited states are eigen-states with even (+) parity under the $C_{2}\left(z\right)$ operator and odd (−) parity under the mirror planes $\sigma_{v}\left(xz\right)$ and $\sigma_{v^{\prime }}\left(yz\right)$. On the other hand, the third and fifth excited states are eigen-states with even (+) parity under all operators. The sequence of vertical excitation energies for the H_2_O molecule is illustrated in Table 5. It’s worth noting that the small discrepancies between the present results and previous GW-BSE studies mainly arise from the different methodologies used to obtain quasi-particle energies and the screened Coulomb interaction. Previous works employed different approaches: Grossman et al. [39] employed a single-shot $G_{0}W_{0}$-BSE approach based on LDA eigenvalues and a plasmon-pole approximation, while Hirose et al. [24] used an all-electron GW-BSE scheme starting from PBE. These approaches are known to be sensitive to the starting point, the treatment of dynamical screening and numerical convergence parameters. Here, we avoid an explicit GW step by using Koopmans’-compliant functionals for the calculation of the quasiparticle energies and by employing linear-response theory to evaluate the static screened Coulomb interaction. We note that the first excited state exhibits $B_{1}$ symmetry. The order of states belonging to the same irreducible representation is given by an integer number on the left of the symmetry label.

**Figure A1:**
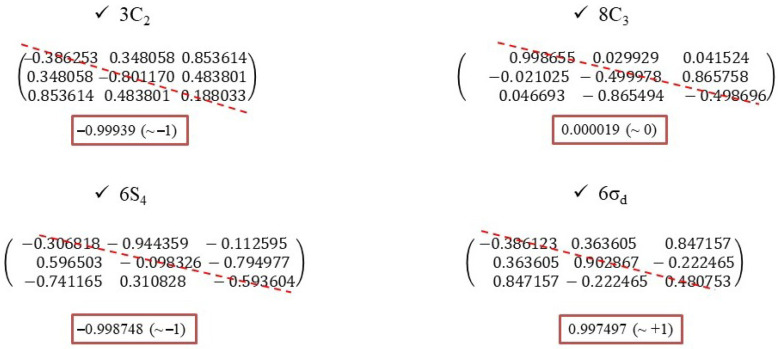
Symmetry matrices for the first excited state of CH_4_ $T_{2}$.

**Figure A2:**
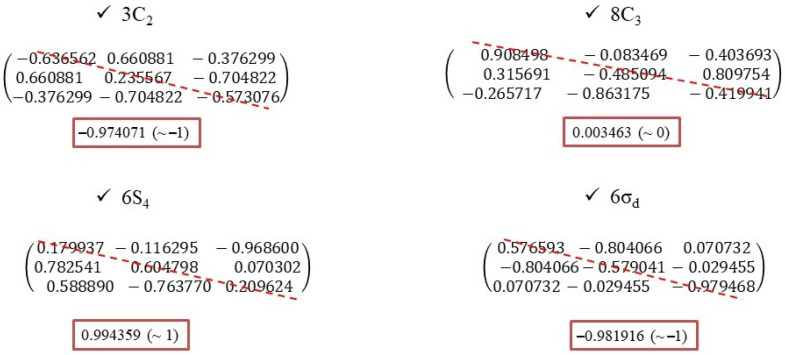
Symmetry matrices for the second excited state of CH_4_ $T_{1}$.

For the CH_4_ molecule, as reported in Table 4, we can observe that all states considered represent multidimensional representations describing degenerate states featuring double or triple degeneracy. Moreover, where states are degenerate, any symmetry operators typically mix degenerate eigenvectors. Therefore, for each degenerate triple subspace (first or second), as well as for double degenerate subspace, we have constructed an m × m matrix (i.e., a 3 × 3 or 2 × 2 matrix) of each symmetry operator restricted to these subspaces, as indicated in Figure A1 and Figure A2. Consequently, the corresponding characters for each sub-group are presented in Table 4. Comparison with the character table (Table 2) of $T_{d}$ reveals that the three first (1–3) and second (4–6) states match the three-dimensional irreducible representations $T_{2}$ and $T_{1}$, respectively. Meanwhile, the first double states (7–8) reveal a two-dimensional irreducible representation, *E*, indicating that the degeneracy originates from the inherent symmetry of the molecule.

In Table 5 and Table 6 we compare the calculated GW-BSE excitation energies with reference theoretical and experimental values. We find that our results are consistent with those obtained in a previous GW-BSE investigations for both molecules. Furthermore, these studies do not explicitly mention the symmetry of the excited states. In contrast, we note that both GW-BSE calculations consistently underestimate the experimental and other theoretical excitation energies for the H_2_O molecule. Whereas, in the case of the CH_4_ molecule, the results obtained with GW-BSE a agree with those achieved with the quantum Monte Carlo (*QMC*) approach, and over estimates the experimental result.

In the next section, we perform a symmetry analysis of the excited states of diatomic molecules, including carbon monoxide (CO) and nitrogen (N_2_). In diatomic molecules, excited states exhibit rotational symmetry relative to the internuclear axis. This symmetry implies that the projection of the orbital angular momentum, ${\hat{L}}_{z}$, onto this axis is a conserved quantity. Consequently, the electronic excited states of a diatomic molecule can be chosen as eigen-functions of ${\hat{L}}_{z}$ with eigen-values ±m. If *m* = 0, the state is labeled Σ (non-degenerate state), whereas |m| = 1, 2, etc. are labeled with capital Greek letters Π, Δ, etc. These means that in diatomic molecules the excited states are either non-degenerate or doubly degenerate. In order to correctly assign the symmetry of doubly degenerate states, we prefer to calculate the expectation values of ${\hat{L}}_{z}^{2}$ which assumes the values $m^{2}=0,1,4,\ldots$ for Σ,Π,Δ,… states, respectively.

Furthermore, the state is also distinguished via the total spin *S* of all electrons. This is given as 2*S* + 1. So that we have a superscript 3 for triplet states and a superscript 1 for singlet states.

In addition, we consider also plane inversion $\sigma_{v}$ for planes containing the molecular axis and, only for homonuclear diatomic molecules, point inversion *I* with respect to the midpoint along the molecular axis. This implies that, relative to plane inversion the excited states have even (+)/odd (−) parity and relative to point inversion (+) or odd (−) parity.The latter are denoted by the subscripts “g” (gerade) and “u” (ungerade), respectively. Thus, the labeling scheme for the electronic state of a diatomic molecule is as follows $L^{2S+1}\Lambda_{\left(g/u\right)}^{\left(\pm \right)}$ with *L* a capital Latin letter specifying the order: A,B,C,…

The symmetry analysis of the excited states of the CO and N_2_ diatomic molecules has been examined with respect to the symmetry operations: angular momentum squared ($L_{z}^{2}$), mirror planes ($\sigma_{v}$), and point inversion for the latter. The results are summarized in Table 7 and Table 8. Furthermore, in case of CO, the first and second states are degenerate, confirming their ^1^Π-type nature. The excited states 3, 6, and 7 are non-degenerate with symmetry ^1^Σ^−^, ^1^Σ^+^, and ^1^Σ^+^, respectively.

For the N_2_ molecule, the results show that the third and sixth states are non-degenerate with symmetry $\Sigma_{u}^{-}$ and $\Sigma_{g}^{+}$, respectively. While the other states are degenerate with $|L_{z}^{2}|$ = 1 or $|L_{z}^{2}|$ = 4, confirming their ^1^Π-type or ^1^Δ-type nature.

The lowest excitation energies obtained for CO and N_2_ are reported in Table 9. From this table, our findings are consistent with those of a previous GW-BSE investigation for both diatomic molecules, CO and N_2_. Nevertheless, the results obtained with both GW-BSE approaches are overestimated compared to the experimental results. Whereas, for CO, GW-BSE reproduces the first excitation energy with an accuracy close to that obtained experimentally. Indeed, in similar systems better results can be achieved only using multi-reference methods.

**Figure 1:**
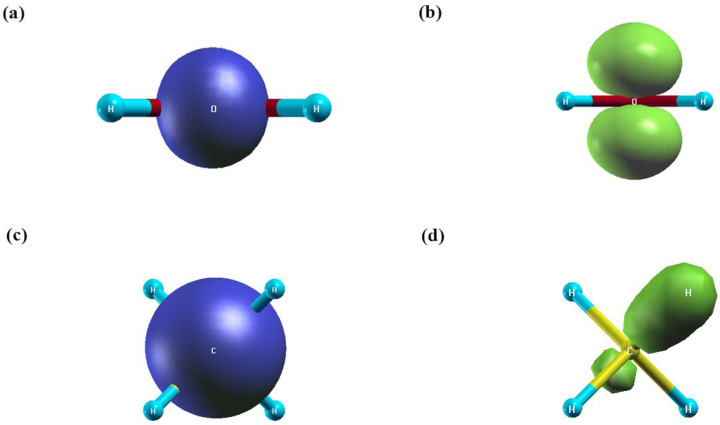
Real-space plots of the excitonic electron and hole wave functions of a heterodiatomic molecule at the GW-BSE level: (**a**,**b**) for water, and (**c**,**d**) for methane. Blue isocontours indicate regions of electron accumulation, whereas green isocontours represent regions of electron depletion.

**Figure 2:**
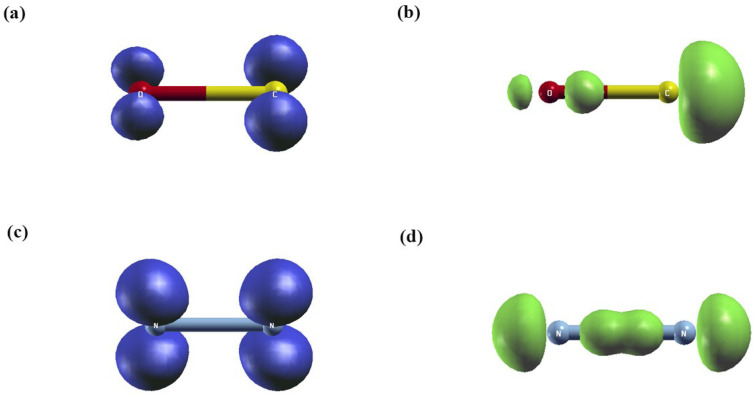
Real-space plots of the excitonic electron and hole densities of a homodiatomic molecule at the GW-BSE level: (**a**,**b**) for carbon monoxide, and (**c**,**d**) for nitrogen. Blue isocontours indicate regions of electron accumulation, whereas green isocontours represent regions of electron depletion.

In order to obtain more insight into the nature and spatial localization of excited states, such as charge transfer or localized excitations, we depicted the real-space analysis of the excitonic hole and electron densities for CO and N_2_ relative to the first excited state, as illustrated in Figure 1 and Figure 2. From the results, it can be observed that the hole density is highly localized on the oxygen atom in H_2_O and around the carbon atom with slight extension toward the hydrogen atoms in CH_4_. This indicates that the removed electron originates mainly from the non-bonding lone-pair orbitals on oxygen in H_2_O and from the C–H bonding orbitals in CH_4_. In contrast, the electron density is centered on the oxygen and carbon atoms and extends along the O-H and C-H bonds toward the hydrogen atoms, emphasizing the delocalized and antibonding character of the excited states.

On the other hand, the excitonic electron and hole densities of the CO molecule are predominantly localized on the oxygen and carbon atoms, respectively. This spatial separation reveals a clear charge-transfer excitation from carbon to oxygen, reflecting the difference in electronegativity between the two atoms. In contrast, for the N_2_ molecule, both the electron and hole densities are symmetrically distributed over the two nitrogen atoms, consistent with its homonuclear and highly symmetric ($D_{\infty h}$) character. Unlike CO, no net charge transfer occurs upon excitation, as the electronic transition is delocalized and equally shared between the two nitrogen atoms.

### 2.2. Fragment

In this section, we perform the symmetry analysis of the excited states of the uracil nucleobase of RNA. The uracil molecule has point group symmetry $C_{s}$, which includes only two symmetry operations, as shown in Table 10: the identity operator *E* and the reflection across the molecular plane σ, as uracil is a flat molecule. Excited states must belong to one of the two A′ and A″ irreducible representations. The symmetry analysis is summarized in Table 11. One can see that the excited states of uracil are eigen-states of the reflection symmetry operator $\sigma_{v}$ with odd (−) or even (+) parity. Furthermore, the sequence of excitation energies for the uracil molecule is A″, A′, A″, A″, and A′, etc., as listed in Table 12.

Table 12 compares our computed vertical excitation energies with previously available experimental and theoretical values. Our results align closely with previous theoretical and experimental findings. For instance, the first and second excited states appear with considerable precision, deviating by less than 0.1–0.3 eV from the experimental values, improving over previous BSE results. Furthermore, the $3^{1}A^{\prime }$ state has been consistently predicted at 6.0 eV, aligning closely with the experimental findings, and being slightly lower than the values obtained with the multi-reference CASPT2 and the best theoretical estimates (BTE-2) reported in Ref. [49].

**Figure 3:**
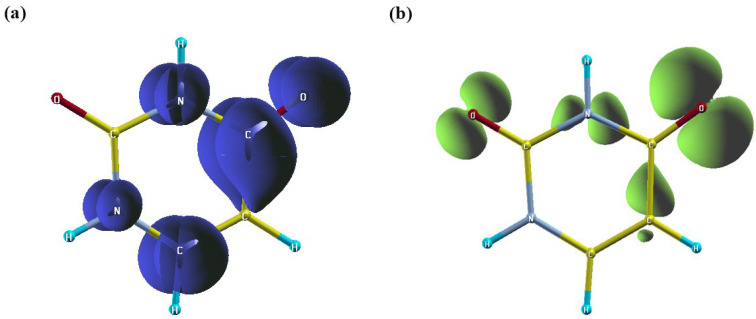
Real-space distributions of the excitonic (**a**) electron and (**b**) hole densities of the uracil nucleobase calculated at the GW-BSE level for the first excited state. Blue isocontours denote regions of electron accumulation, while green isocontours indicate regions of electron depletion.

On the other hand, to better understand the nature and spatial distribution of the excited states in uracil, the excitonic hole and electron densities relative to the first excited state were analyzed in real space and are shown in Figure 3. It can be seen that the excitonic electron density is mainly localized in the C=O bond region of uracil and slightly extends toward the adjacent C-N bond, consistent with the population of the antibonding $\pi^{*}$ orbital of the carbonyl group. In contrast, the corresponding hole density is predominantly localized on the oxygen atom, indicating that an electron has been removed from a nonbonding (n) orbital. Our results are in excellent agreement with previous theoretical investigations, particularly those employing wave-function-based methods such as CC2 and CCSD, which consistently report the same state assignments and qualitative electronic character for the low-energy excited states of uracil [54].

These observations clearly demonstrate that the first excited state possesses a localized character.

To gain deeper insight into these spatial features, we employ the concept of local symmetry. In the context of quantum chemistry, local symmetry denotes the spatial characteristics of excitations that are confined to specific regions of a molecule. Unlike the overall molecular symmetry, local symmetry represents an approximate or fragmental symmetry associated with particular bonds, functional groups, or subunits of the molecular structure. As mentioned above, the uracil nucleobase has relatively low global molecular symmetry ($C_{s}$) because its molecular structure is planar but not perfectly symmetric across all atoms. Therefore, when analyzing the first excited state, the excitonic wavefunction (or electron-hole pair) is primarily localized on the C=O bond. This implies that this part of the molecule has a local environment that closely resembles the point group $C_{2v}$, which has the same symmetry as the H_2_O molecule, discussed previously.

As the first excited state is localized on the C=O bond we want to analyze it in terms of the local $C_{2v}$ symmetry. For this goal, we first obtain the local excitation through the procedure described in the Theory Section. Then we computed the expectation values of the $C_{2}$, $\sigma_{v}$ and $\sigma_{v}^{\prime }$ operators. These yield 0.485, −0.477 and −0.940, respectively. This means, from a comparison with the character table (Table 1) of $C_{2v}$, that locally the first excited state displays a partial $A_{2}$ symmetry.

## 3. Materials and Methods

The structure of most small molecules, defined as the position of their atoms, displays invariance with respect to one or more symmetry operations. In the three dimensional euclidean space, such operations are: *E* identity, $C_{n}$ n-fold rotation abut an axis, σ reflection about a plane, *i* inversion about a point, $S_{n}$ n-fold rotation abut an axis followed by a reflection about a plane perpendicular to the axis (improper rotation). In case of axial molecules as diatomic molecules we should include rotations $C_{\infty }$ and improper rotations $S_{\infty }$ of an arbitrary angle [55]. The set of symmetry operations which leave invariant the structure of a molecule defines its point group. Symmetry operations commute with the electronic many-body Hamiltonian operator. If applied to a many-electrons eigen-state of the molecule, one of its symmetry operation yields an eigen-state with the same energy. Hence, non-degenerate states will be transformed in the same state but a sign (1 or −1), whereas degenerate states will be transformed in a linear combination of them. This means that states with the same energy form a basis for representing the symmetry operations of the molecule. The traces of the resulting matrices gives a fingerprint for identifying which is the particular irreducible representation looking to the character table.

For building such matrices we have first to look at the form of the BSE many-body states. We consider here the case the staring DFT calculation has $N_{v}$ doubly occupied valence states. The many-body ground-state $\Psi_{0}$ is written as:

(1) $$\Psi_{0}\left(x_{1},\ldots ,x_{2N_{v}}\right)=\frac{1}{\sqrt{(2N_{v})!}}\det\left|\chi_{1,\alpha }\left(x_{1}\right)\chi_{1,\beta }\left(x_{2}\right)\ldots \chi_{N,\alpha }\left(x_{2N-1}\right)\chi_{N,\beta }\left(x_{2N}\right)\right|$$

where we indicate with x=(r,ω) the general space and spin coordinates, $\chi_{i,\alpha }\left(x\right)=\phi_{i}\left(r\right)\alpha \left(\omega \right)$, $\phi_{i}$ is the *i*-th DFT orbitals of energy $\epsilon_{i}$ and α(ω), β(ω) the two collinear spin states, eigen-states of ${\hat{s}}_{z}$. In the det we report only the first line. For sake of simplicity we use the second quantization picture. Within the Tamm-Dancoff approximation, a triplet excited states, is written, accordingly to the $m_{s}$ axial spin ${\hat{S}}_{z}$, is written as:

(2) $$\begin{array}{cc} |\Psi^{3,m_{s}=1}\rangle & =-\sum_{vc}A_{vc}{\hat{a}}_{c\alpha }^{†}{\hat{a}}_{\beta } \end{array}$$

(3) $$\begin{array}{cc} |\Psi^{3,m_{s}=0}\rangle & =\frac{1}{\sqrt{2}}\sum_{vc}A_{vc}\left({\hat{a}}_{c\alpha }^{†}{\hat{a}}_{v\alpha }-{\hat{a}}_{c\beta }^{†}{\hat{a}}_{v\beta }\right)|\Psi_{0}\rangle \end{array}$$

(4) $$\begin{array}{cc} |\Psi^{3,m_{s}=-1}\rangle & =\sum_{vc}A_{vc}{\hat{a}}_{c\beta }^{†}{\hat{a}}_{v\alpha } \end{array}$$

while a singlet state has the form:

(5) $$|\Psi^{1}\rangle =\frac{1}{\sqrt{2}}\sum_{vc}A_{vc}\left({\hat{a}}_{c\alpha }^{†}{\hat{a}}_{v\alpha }+{\hat{a}}_{c\beta }^{†}{\hat{a}}_{v\beta }\right)|\Psi_{0}\rangle$$

In the so-called batches representation [6,19,56], used in TD-DFT and BSE for avoiding explicit reference to and evaluation of empty DFT orbitals. This is a major issue in ordinary BSE and TD-DFT approaches as, in principles, the number of empty orbitals equals the dimension of the basis used to represent them. The idea is to define formally the batches as:

(6) $$\xi_{v}\left(r\right)=\sum_{c}A_{vc}\phi_{c}\left(r\right)$$

Then, inserting this equation in the BSE formalism it is possible to derive a formulation [6,19] where only the $\xi_{v}\left(r\right)$’s functions appear without any reference to the *c*-index. The $\xi_{v}\left(r\right)$ are treated as one-particle wave-functions constrained to belong to the manifold of the empty DFT orbitals. In order to enforce this, we make use of the projector operator ${\hat{P}}_{c}$ on the empty manifold. In turn, this is expressed, using the so-called resolution of the identity, in terms of the occupied, valence, KS orbitals:

(7) $${\hat{P}}_{c}=1-\sum_{v}|\phi_{v}\rangle \langle \phi_{v}|$$

A set of $N_{v}$ $\xi_{v}$’s functions, one for each valence state *v*, is required to represent an excited state. The normalization conditions for both $\Psi^{3}$ and $\Psi^{1}$ are written as:

(8) $$\sum_{v}\langle \xi_{v}|\xi_{v}\rangle =1$$

this implies that the single $\xi_{v}$ are not normalized. Therefore, we prefer to define the normalized vectors:

(9) $$|{\tilde{\xi }}_{v}\rangle =\frac{|\xi_{v}\rangle }{\sqrt{\langle \xi_{v}|\xi_{v}\rangle }}$$

This permits us to write $\Psi^{1}$ explicitly as a sum of determinants:

(10) $$\begin{array}{cc} |\Psi^{1}\rangle & =\frac{1}{\sqrt{2(2N)!}}\sum_{v=1,N_{v}}A_{vc}\det\left|\chi_{1,\alpha }\chi_{1,\beta }\ldots \chi_{c,\alpha }\chi_{v,\beta }\ldots \chi_{N_{v},\alpha }\chi_{N_{v},\beta }\right|\\ \; & +\\ \; & \frac{1}{\sqrt{2(2N)!}}\sum_{v=1,N_{v}}A_{vc}\det\left|\chi_{1,\alpha }\chi_{1,\beta }\ldots \chi_{v,\alpha }\chi_{c,\beta }\ldots \chi_{N_{v},\alpha }\chi_{N_{v},\beta }\right| \end{array}$$

summing over *c* and using Equations (6) and (9), we get:

(11) $$\begin{array}{cc} |\Psi^{1}\rangle & =\frac{1}{\sqrt{2(2N)!}}\sum_{v=1,N_{v}}\sqrt{\langle \xi_{v}|\xi_{v}\rangle }\det\left|\chi_{1,\alpha }\chi_{1,\beta }\ldots {\tilde{\xi }}_{v,\alpha }\chi_{v,\beta }\ldots \chi_{N_{v},\alpha }\chi_{N_{v},\beta }\right|\\ \; & +\\ \; & \frac{1}{\sqrt{2(2N)!}}\sum_{v=1,N_{v}}\sqrt{\langle \xi_{v}|\xi_{v}\rangle }\det\left|\chi_{1,\alpha }\chi_{1,\beta }\ldots \chi_{v,\alpha }{\tilde{\xi }}_{v,\beta }\ldots \chi_{N_{v},\alpha }\chi_{N_{v},\beta }\right| \end{array}$$

with the definitions ${\tilde{\xi }}_{v,\alpha }\left(x\right)={\tilde{\xi }}_{v}\left(r\right)\alpha \left(\omega \right)$ and ${\tilde{\xi }}_{v,\beta }\left(x\right)={\tilde{\xi }}_{v}\left(r\right)\beta \left(\omega \right)$. We get analogous expressions fo the $\Psi^{3}$’s. For example, in the case of $\Psi^{3,m=1}$, we get:

(12) Ψ3,m=1=1(2N)!∑v=1,Nv〈ξv|ξv〉detξ~v,αχ1,αχ1,β…χv,αχv,β…χNv,αχNv,β

The evaluation for a set of degenerate excited states of the character table requires the computation of the (small) matrices:

(13) $$S_{ij}=\langle \Psi_{i}^{1}|\hat{S}|\Psi_{j}^{1}\rangle$$

and

(14) $$S_{ij}=\langle \Psi_{i}^{3,m_{s}=1,0,-1}|\hat{S}|\Psi_{j}^{3,m_{s}=1,0,-1}\rangle$$

where $\Psi^{3}$ is one of the three triplet states and *i* and *j* run over a set of degenerate singlet or triplet states and $\hat{S}$ is a symmetry operator. As the application of $\hat{S}$ on one-particle wavefunctions is straightforward, we introduce the notation:

(15) $$\begin{array}{cc} |\phi_{v}^{S}\rangle & =\hat{S}|\phi_{v}\rangle \\ |{\tilde{\xi }}_{v}^{S}\rangle & =\hat{S}|{\tilde{\xi }}_{v}\rangle \end{array}$$

As the calculation of Equation (13) requires the evaluation of the scalar product of several couples of Slater’s determinants, we calculate first all the one-particle terms:

(16) $$\begin{array}{cc} O_{vv^{\prime }}^{VV} & =\langle \phi_{v}|\phi_{v^{\prime }}^{S}\rangle \\ O_{vv^{\prime }}^{VC} & =\langle \phi_{v}|\xi_{v^{\prime }}^{(j),S}\rangle \\ O_{vv^{\prime }}^{CV} & =\langle \xi_{v}^{\left(i\right)}|\phi_{v^{\prime }}^{S}\rangle \\ O_{vv^{\prime }}^{CC} & =\langle \xi_{v}^{\left(i\right)}|\xi_{v^{\prime }}^{(j),S}\rangle \end{array}$$

where with an (i) superscript we indicate quantities related to the *i*-th exciton. The scalar product than can be obtained in following way. First, we reorder the columns of the Slater’s determinants in order to group α and β spin components, as in:

(17) Ψ3,ms=1=1(2N)!∑v=1,Nv〈ξv|ξv〉(−1)Nv(Nv−1)/2+Nvdetχ1,αχ2,α…χNv,αξ~v,αχ1,βχv,β…χNv,β

Then, we use the property telling that the scalar product of two Slater’s determinants equals the determinant of the scalar products of the orbitals together with the property:

(18) $$\det\left|\begin{array}{cc} A & 0\\ 0 & B \end{array}\right|=\det A\det B$$

with *A* and *B* square matrices. This yields:

(19) $$\begin{array}{cc} \langle \Psi_{i}^{3,m_{s}=1}|\hat{S}|\Psi_{j}^{3,m_{s}=1}\rangle & =\frac{1}{(2N)!}\sum_{v,v^{\prime }}\sqrt{\langle \xi_{v}^{\left(i\right)}|\xi_{v}^{\left(i\right)}\rangle }\sqrt{\langle \xi_{v^{\prime }}^{\left(j\right)}|\xi_{v^{\prime }}^{\left(j\right)}\rangle }\det M_{\left(v,v^{\prime }\right)}^{\left(i,j\right)}\det{\bar{M}}_{\left(v,v^{\prime }\right)}^{\left(i,j\right)} \end{array}$$

where $M_{\left(v,v^{\prime }\right)}^{\left(i,j\right)}$ and ${\bar{M}}_{\left(v,v^{\prime }\right)}^{\left(i,j\right)}$ are matrices of order N+1 and N−1, respectively. They read:

(20) $$\begin{array}{cc} M_{(v,v^{\prime }),\gamma \delta }^{\left(i,j\right)} & =\left\{\begin{array}{cc} O_{\gamma \delta }^{VV} & \gamma ,\delta \leq N\\ O_{\gamma v^{\prime }}^{VC} & \gamma \leq N,\delta =N+1\\ O_{v\delta }^{CV} & \gamma =N+1,\delta \leq N\\ O_{vv^{\prime }}^{CC} & \gamma =N+1,\delta =N+1 \end{array}\right. \end{array}$$

and

(21) $$\begin{array}{cc} {\bar{M}}_{(v,v^{\prime }),\gamma \delta }^{\left(i,j\right)} & =\left\{\begin{array}{cc} O_{\gamma \delta }^{VV} & \gamma <v,\delta <v^{\prime }\\ O_{\gamma +1\;\delta }^{VV} & \gamma \geq v,\delta <v^{\prime }\\ O_{\gamma \delta +1}^{VV} & \gamma <v,\delta \geq v^{\prime }\\ O_{\gamma +1\;\delta +1}^{VV} & \gamma \geq v,\delta \geq v^{\prime } \end{array}\right. \end{array}$$

While, for singlet states, the scalar product than can be written as:

(22) $$\begin{array}{cc} \langle \Psi_{i}^{1}|\hat{S}|\Psi_{j}^{1}\rangle & =\frac{1}{(2N)!}\sum_{v,v^{\prime }}\sqrt{\langle \xi_{v}^{\left(i\right)}|\xi_{v}^{\left(i\right)}\rangle }\sqrt{\langle \xi_{v^{\prime }}^{\left(j\right)}|\xi_{v^{\prime }}^{\left(j\right)}\rangle }\det P_{\left(v,v^{\prime }\right)}^{\left(i,j\right)}\det{\bar{P}}_{\left(v,v^{\prime }\right)}^{\left(i,j\right)} \end{array}$$

here $P_{\left(v,v^{\prime }\right)}^{\left(i,j\right)}$ and ${\bar{P}}_{\left(v,v^{\prime }\right)}^{\left(i,j\right)}$ are matrices of order *N* which read:

(23) $$\begin{array}{cc} P_{(v,v^{\prime }),\gamma \delta }^{\left(i,j\right)} & =\left\{\begin{array}{cc} O_{\gamma \delta }^{VV} & \gamma \neq v,\delta \neq v^{\prime }\\ O_{\gamma v^{\prime }}^{VC} & \gamma \neq v,\delta =v^{\prime }\\ O_{v\delta }^{CV} & \gamma =v,\delta \neq v^{\prime }\\ O_{vv^{\prime }}^{CC} & \gamma =v,\delta =v^{\prime } \end{array}\right. \end{array}$$

and

(24) $$\begin{array}{cc} {\bar{P}}_{(v,v^{\prime }),\gamma \delta }^{\left(i,j\right)} & =O_{\gamma \delta }^{VV} \end{array}$$

When analyzing the properties of the excited states of a system symmetric with respect to arbitrary rotations around an axis *x*, it is convenient to evaluate the expectation value of the squared angular momentum operator ${\hat{L}}_{x}^{2}$. We recall that the operator ${\hat{L}}_{x}$ applied on generic Slater’s determinant $\det\phi_{1}\ldots \phi_{N}$ yields:

(25) $${\hat{L}}_{x}\det\left|\phi_{1}\ldots \phi_{N}\right|=\det\left|\left({\hat{L}}_{x}\phi_{1}\right)\phi_{2}\ldots \phi_{N}\right|+\det\left|\phi_{1}\left({\hat{L}}_{x}\phi_{2}\right)\ldots \phi_{N}\right|+\ldots +\det\left|\phi_{1}\phi_{2}\ldots \left({\hat{L}}_{x}\phi_{N}\right)\right|$$

In case of large, possibly nano-structured, systems, the excitations associated with BSE excited states are spatially localized. This happens, for example, in optically active defects of bulk materials. We can define *reduced* singlet or triplet excited state $\left.|\Psi^{(1,3),loc}\right.\rangle$ accounting solely for the valence orbitals involved in the excitation that is originally described by a regular excited state $\left.|\Psi^{\left(1,3\right)}\right.\rangle$. The number of batches of $\left.|\Psi^{(1,3),loc}\right.\rangle$ is given by the number of batches of the departing states which satisfy $\langle \xi_{v}|\xi_{v}\rangle >\sigma$ with σ an opportune threshold. Then, the valence orbitals and batches of $\left.|\Psi^{(1,3),loc}\right.\rangle$ are those of $\left.|\Psi^{\left(1,3\right)}\right.\rangle$ passing the same criteria. Finally, we renormalize $\left.|\Psi^{(1,3),loc}\right.\rangle$. This allows for the study of the properties of a localised excitation in terms of the local structural symmetry.

## 4. Conclusions

We have designed and implemented a method for analyzing the symmetry properties of excited states calculated within the GW-BSE approach. In case of small molecules, this lead to a prompt assignment to irreducible representation and correct labeling. We have also shown the states, if localized, can be analyzed in terms of the symmetry of then local structure. This could be used for addressing localized defect states in crystals as those on focus for hosting q-bits [57]. Our method could also be useful for investigating chiral states in molecules [58]. Our approach can promptly be implemented in TD-DFT codes as those based on the so-called resolution of the identity for avoiding sums over empty states [56,59]. Our approach can also be used for differentiating among almost degenerate states thanks to its negligible numerical errors. Moreover, it could be applied also to other operators embodying different observables as, for examples, dipole moments.

## Figures and Tables

**Table 1:** Character table of the $C_{2v}$ symmetry group for the H_2_O molecule.

$C_{2v}$	*E*	$C_{2}\left(z\right)$	$\sigma_{v}\left(\mathrm{xz}\right)$	$\sigma_{v^{\prime }}\left(\mathrm{yz}\right)$
$A_{1}$	1	1	1	1
$A_{2}$	1	1	−1	−1
$B_{1}$	1	−1	1	−1
$B_{2}$	1	−1	−1	1

**Table 2:** Character table of the T*_d_* symmetry group for the CH_4_ molecule.

T_*d*_	E	8C_3_	3C_2_	6S_4_	6$\sigma_{d}$
A_1_	1	1	1	1	1
A_2_	1	1	1	−1	−1
E	2	−1	2	0	0
T_1_	3	0	−1	1	−1
T_2_	3	0	−1	−1	1

**Table 3:** Symmetry analysis and labeling of excited states for H_2_O.

Excited State	$C_{2}\left(z\right)$	$\sigma_{v}\left(\mathrm{xz}\right)$	$\sigma_{v^{\prime }}\left(\mathrm{yz}\right)$	Label
1	−0.9949	0.9949	−0.9999	$1^{1}B_{1}$
2	0.9946	−0.9947	−0.9999	$1^{1}A_{2}$
3	0.9981	0.9981	0.9999	$1^{1}A_{1}$
4	−0.9988	0.9988	−0.9999	$2^{1}B_{1}$
5	0.9978	0.9978	0.9999	$2^{1}A_{1}$
6	−0.9967	−0.9968	0.9999	$1^{1}B_{2}$
7	−0.9996	0.9996	−0.9999	$3^{1}B_{1}$
8	0.9979	−0.9980	−0.9999	$2^{1}A_{2}$

**Table 4:** Symmetry analysis and labeling of excited states for CH_4_.

Excited State	$8C_{3}$	$3C_{2}$	$6S_{4}$	$6\sigma_{d}$	Label
1					
2	0.00001	−0.9993	−0.9987	0.9974	$T_{2}$
3					
4					
5	0.0034	−0.9740	0.9943	−0.9819	$T_{1}$
6					
7	−0.9875	1.9601	0.0095	0.0103	*E*
8					

**Table 5:** Excitation energies for H_2_O. All results are in eV.

State	$1^{1}B_{1}$	$1^{1}A_{2}$	$1^{1}A_{1}$	$2^{1}B_{1}$	$2^{1}A_{1}$	$1^{1}B_{2}$	$3^{1}B_{1}$	$2^{1}A_{2}$
This work (GW-BSE)	6.534	7.762	8.889	10.152	10.487	10.756	11.231	11.255
GW-BSE [24]	6.43	8.35	–	–	–	–	–	–
CASSCF [40]	7.40	9.09	–	–	–	–	–	–
CCSD [40]	7.59	9.35	–	–	–	–	–	–
Exp [41]	7.4	9.1	–	–	–	–	–	

**Table 6:** Excitations energies for CH_4_. All results are in eV.

State	$T_{2}$	$T_{1}$	*E*
This work (GW-BSE)	10.471	11.019	11.257
GW-BSE [39]	10.5	–	–
QMC [39]	10.4	–	–
Exp [42]	8.52	–	–

**Table 7:** Symmetry analysis and labels of excited states for CO molecule.

Excited State	$|L^{2}|$	$\sigma_{v}$	Label
1	1.0024	−0.1072	*A*^1^Π
2	1.0024	0.1071	*A*^1^Π
3	0.0025	−0.9997	*B*^1^Σ^−^
4	4.0013	−0.9968	*C*^1^Δ
5	4.0013	0.9968	*C*^1^Δ
6	0.0188	0.9997	*D*^1^Σ^+^
7	0.0127	0.9997	*E*^1^Σ^+^
8	1.0267	0.1516	*F*^1^Π

**Table 8:** Symmetry analysis and labels of the excited states of N_2_.

Excited State	$|L^{2}|$	*I*	$\sigma_{v}$	Label
1	1.0015	0.9996	−0.9996	$A^{1}\Pi_{g}$
2	1.0015	0.9996	−0.9996	$A^{1}\Pi_{g}$
3	0.0015	−0.9999	−0.9999	$B^{1}\Sigma_{u}^{-}$
4	4.0014	−0.9998	−0.9998	$C^{1}\Delta_{u}$
5	4.0014	−0.9998	−0.9998	$C^{1}\Delta_{u}$
6	0.0136	0.9986	0.9986	$D^{1}\Sigma_{g}^{+}$
7	1.0175	−0.9994	0.9994	$E^{1}\Pi_{u}$
8	1.0163	−0.9994	0.9994	$E^{1}\Pi_{u}$

**Table 9:** Excitation energies for CO and N_2_. All results are in eV.

State	*A*^1^Π	*B*^1^Σ^−^	*C*^1^Δ	*D*^1^Σ^+^	*E*^1^Σ^+^	*F*^1^Π
CO						
This work (GW-BSE)	7.995	9.048	9.725	9.949	10.813	10.957
GW-BSE [24]	7.67	8.24	–	–	–	–
CASSCF-icMRCI [43]	8.14	8.19	8.48	–	–	–
Exp [44]	8.07	8.07	8.17	–	–	–
**State**	$A^{1}\Pi_{g}$	$B^{1}\Sigma_{u}^{-}$	$C^{1}\Delta_{u}$	$D^{1}\Sigma_{g}^{+}$	$E^{1}\Pi_{u}$	
**N_2_**						
This work (GW-BSE)	8.615	8.823	9.691	11.194	11.992	
GW-BSE [24]	7.93	8.29	–	–	–	
EOM-CCSDT [45]	9.681	9.155	10.067	–	–	
Exp [46]	9.31	9.97	–	–	–	

**Table 10:** Character table of $C_{s}$ symmetry group for the uracil nucleobase.

$C_{s}$	*E*	σ
$A^{\prime }$	1	1
$A^{\prime \prime }$	1	−1

**Table 11:** Symmetry analysis and labels of the lowest excited states in uracil nucleobase.

Excited State	$\sigma_{v}$	Label
1	−0.9998	$1^{1}A^{\prime \prime }$
2	0.9998	$2^{1}A^{\prime }$
3	−0.9998	$2^{1}A^{\prime \prime }$
4	−0.9999	$3^{1}A^{\prime \prime }$
5	0.9994	$3^{1}A^{\prime }$
6	−0.9993	$4^{1}A^{\prime \prime }$
7	−0.9999	$5^{1}A^{\prime \prime }$
8	−0.9999	$6^{1}A^{\prime \prime }$
9	0.9998	$4^{1}A^{\prime }$
10	0.9998	$5^{1}A^{\prime }$

**Table 12:** Comparison of singlet excitation energies (in e*V*) for uracil nucleobase obtained with GW-BSE.

State	1^1^*A*″	2^1^*A*′	2^1^*A*″	3^1^*A*″	3^1^*A*′	4^1^*A*″	4^1^*A*′	5^1^*A*′
This work (GW-BSE)	4.277	5.159	5.314	5.845	6.006	6.120	6.422	6.584
KI-BSE [7]	3.96	5.38	5.96	–	6.04	–	–	–
TD-PBE0 [47]	4.73	5.30	5.94	6.36	6.06	–	6.66	–
$G_{0}W_{0}$-BSE [48]	3.80	4.43	5.02	5.52	5.17	–	5.89	–
BTE-2 [49]	5.00	5.25	6.10	6.56	6.26	–	6.70	–
CASPT2 [50]	4.93	5.23	6.37	7.22	6.17	6.94	6.69	7.33
Exp [51]	4.38 [52] 4.9–5.2 [53]	5.1			6.0		6.6	6.9–7.0

## Data Availability

The original contributions presented in this study are included in the article. Further inquiries can be directed to the corresponding author.

## Abbreviations

The following abbreviations are used in this manuscript:

DFT	Density Functional Theory
BSE	Bethe-Salpeter Equation

## Appendix A

The following analysis summarizes the exciton degeneracies obtained from the symmetry matrices, which were constructed using the BSE output for the CH_4_ molecule.
